# The Cure of Rupture

**Published:** 1877-08

**Authors:** 


*The cure of Rupture reducable and irreducable, also of Varicoele and Hydrocele*, by new Methods. By GEOREG HEATON, M. D., Member Mass. Med. Society ; Fellow of the Royal Chirurgical Society of London ; of the Westminster and London Med. Societies; of the Parisian Med. Society of France, etc. Arranged and Edited by J. Henry Davenport, A. M., M. D.

Dr. Heaton does not say that for about a quarter of a century he has offered radical cure of Hernia by an advertisement in the Boston Medical Journal, and that he has never made any impression upon the profession, or in the least changed or influenced the opinions of Surgeons as to the feasibility, safety or truth, of reported cases of radical cure, He does not say that he offered as much twenty-five years ago as now, but calls his operation new.

We propose giving a quotation from the Author, and hope our readers will judge for themselves of this very important matter of radical cure of Hernia, by the "method of *tendinous irritation*," which we confess looks to us "pretty thin."

"It is worthy of observation that all of the operations which have been at varions times proposed for the cure of hernia may well be classed among the severe, if not violent, procedures of surgery. Their performance is always succeeded by more or less inflammation, which is often quite extensive, and in some cases is uncontrollable and dangerous. In Gerdy's, Wutzer's, and Wood's opepations, which, though now discarded, obtained in their day considerable notice, an attempt was made to close the hernial opening with a plug formed either of the adjacent integument or of the invaginated scrotal tissues or of both, in expectation that adhesions would form sufficient to retain this rude plug in position and thus prevent the descent of the protrusion.

But even when the violence of the inflammation thus usually excited has subsided and afforded the surgeon an apparent success, the tendency of nature is to get rid of these tissues, which are like foreign bodies in a canal, and the rupture usually soon recurs. But I have alluded to the operations only to remark upon the feature which is common to them all, and which seems to me the true reason why they fail to cure, namely, the excessive and generally suicidal degree of inflammation produced by them one and all. This is the reason why they have not been and never should be generally received as accredited operations; and the same remark applies to my own early operations for radical cure by means of injection of the essential oils. My attention was first drawn to this by observing with some surprise that whenever in my operations the early result was seemingly unsatisfactory because of too little apparent effect, it proved the best as to permanent cure, and I was thus led to persist in efforts which at first were thought likely to prove utterly futile.

I lay it down, therefore, as a cardinal principle in all operations for the cure of hernia, that any inflammation except of the mildest grade must be carefully avoided. If the surgeon cannot operate without producing the four cardinal principles of inflammation described by Celsus, namely, heat, pain, redness, and swelling, he had much better let his patient alone, and not do harm, for such a result cannot do good.

"It is important to avoid inflammation not only because its occurrence weakens if not utterly prevents the alterations we aim to produce by operating, but also because it compels an early abandonment of the means we make use of to support the protrusion during the process of cure. If inflammation is once allowed to occur, the truss or bandage which should be worn for a time will cause much discomfort, and must of necessity be removed. In fact, the patient himself will soon take this off if the surgeon does not, and thus all possibility of a cure will be prevented at the outset.

"Gradually convinced of the truth of the above considerations, for a long time I patiently sought for a practical means of accomplishing this desired avoidance of inflammation, and after eight years of discouraging experiment discovered a process which I call *the method of tendinous irritation.* This form of treatment I have now prarticed for many years and in many hundreds of cases, so that I am now well convinced of its value. It may be briefly described as consisting of a mild irritation of those portions of fibrous tissue lying directly in contact with the exterior of the neck of the hernial sac, thickening and consolidating their substance, and effecting a contraction of the openings. This contraction is due not only to the astringent used as an irritant but also largely to the peculiar normal distribution of the bundles of fibre in the neighborhood of the abdominal rings. Below are given two modes of operation, by either of which it is possible to effect a cure, and I myself use them indifferently.

"*Operation for Radical Cure.* (Liquid method or method of injection.*) The operator must be provided with an instrument somewhat resembling the ordinary subcutaneous syringe, which is loaded with the modicum of irritating fluid. This instrument as well as the special form of irritant used will be fully described below. The patient should be placed on a bed in the recumbent position, and the contents of the hernia returned within the abdomen. The position of the patient then usually suffices to prevent the return of the protrusion while operating. If not, it should be retained by the finger of an assistant. The hernial sac also should then be returned to the abdomen if possible. It is true that in the majority of cases this cannot be done, yet the effort should always be made in order to empty the canal as much as possible. If it cannot be returned, the sac may be allowed to remain in its accustomed position, as its presence in the canal only somewhat diminishes the effect of

* This operation, though it may be termed the operation by injection, should not be confounded with the operation of injection alluded to in many text-books of surgery, which consists of an injection of the *interior of the hernial sac* with tincture of iodine or some other inflammatory agent.

the operation, but not by any means actually prevents an entirely satisfactory result. Armed with the instrument, which is supposed to be charged and prepared, the operator introduces its beak into the inguinal canal, but outside of the sac, if this has beeen suffered to remain in the following manner: invaginate the right forefinger in the scrotum and find the external abdominal ring, then with the left forefinger press perpendicularly upon the integument directly over this ring, and use sufficient force to, if possible, press the integument together with the finger directly into the riug. The left forefinger being at or in the ring, the spermatic cord and the sac, if in the way, are to be pushed to one side so that nothing may remain between the external pillar of the ring and the finger except the integument and subjacent superficial fasciæ. Keeping the left forefinger thus, take the instrument in the right hand and introduce its freshly sharpened and polished beak *quickly*, penetrating the integument and superficial fasciæ, just passing but not grazing the external pillar, and entering the canal at once. Then remove the left forefinger and gently insinuate the beak further on, well into the canal, exercising the greatest care not to impinge upon the spermatic cord, which is sensitive to tne slighest touch, or upon the fibrous walls of the canal. To wound any of these parts endangers the success of the operation, and to penetrate the transversalis fascia would be particularly unfortunate. If the operator in attempting to pass through the ring should impinge upon or transfix one of the pillars (an accident to which the tyro is very liable), the instrument will not be able to be freely and easily moved about, which is to a remarkable extent when the canal is successfully entered. But before proceeding any further the surgeon may do well to confirm his diagnosis of position by transferring the instrument to the left hand, while with the right forefinger invaginated in the scrotal tissues he explores the inguinal region, and examines the exact situation of the beak. Beyond the prick of the puncture the patient suffers but little pain if the introduction is skillfully performed. But any awkward movements of the beak about the spermatic cord will cause sharp pain, which is referred to the testicle or to the deeper parts of the abdomen.

"Having satisfied himself that the beak of his instrument is in the canal, the surgeon then deposits about ten minims of the liquid irritant, emitting it drop by drop and spreading it as much as possible. The beak of the instrument should be well swept about while delivering its contents, passing around the exterior of the sac if unreduced and wetting all the fibrous tissues. Particular care should be taken that the intercolumnar or arciform fibres and the inner edges of the external ring are wet with the irritant. The canal is usually found much more free than would be anitcipated, and any adventitious adhesions can be either broken or avoided. A small though essential amount of the irritant should be placed in the extreme upper portion of the canal, so as to operate upon the fibres embracing the internal abdominal ring. Owing not only to its proximity to the abdomen, but also, and more especially, to the usual presence in the upper part of the canal of a few muscular fibres of the internal oblique, the sensitiveness to irritation here is extreme and the slightest amount of material produces all the effect that is usually desirable.

"Having wet the entire fibrous interior of the canal and of the inguinal rings, the beak is then withdrawn quickly so that none of the injection may be left in the cellular tissue and fasciæ lying beneath the integument and just exterior to the external abdominal ring. At the instant of withdrawing the beak press the finger over the puncture, thus preventing any oozing of blood which might occur if the skin is delicate, and also in the case of a hernia with a free opening hindering any of the injection which has not been absorbed from oozing outward. The application of the irritant may cause some slight immediate pain, which is soon allayed by the morphine which is contained in the injection. The previous protrusion should not be allowed to descend after the application of the irritant, nor the patient be permitted to assume even the sitting position, until a suitable bandage or means of support has been properly applied.

"*The Irritant.* Take of Thayer's Fluid Extract of Quercus Alba, prepared in vacuo, one half an ounce; of the solid alcoholic Extract of Quercus Alba, about fourteen grains. Triturate with the aid of gentle heat for a long time in a mortar until the solution is as perfect as possible. It is well not to exceed this amount of the solid extract, else the mixture will be too irritating. I usually prepare a quantity of this mixture sufficient for six month's or a year's supply, and am very cautious in first using it, adding a little more of the solid or the fluid extract, accordingly as I observe it ptoduces to little or to great an effect. Having once adjusted the proportions in this manner, and satisfactorily tested the mixture, I use it and no other until the supply is exhausted. The proportions never need vary much from what is stated above. In preparing a new mixture I always get the extracts fresh from the laboratory, and am particular to procure fluid extract which has been concentrated *in vacuo.*

"Of late years it has also been my habit to add to this mixture the sulphate of morphine in the proportion of about one grain to the ounce. This has the effect of diminishing the dull aching that follows the operation, which is caused by the irritation of tendinous tissue. It also serves the further purpose of constipating the bowels, which is also induced by the tannin in the mixture. The amount of this mixture used at any one operation is, as said before, about ten minims."

